# Active screening of patients with diabetes mellitus for pulmonary tuberculosis in a tertiary care hospital in Sri Lanka

**DOI:** 10.1371/journal.pone.0249787

**Published:** 2021-04-08

**Authors:** Sumudu Hewage, Noel Somasundaram, Vithiya Ratnasamy, Ishara Ranathunga, Amitha Fernando, Indika Perera, Udara Perera, Dhammika Vidanagama, Mizaya Cader, Poorna Fernando, Nirupa Pallewatte, Lakmal Rathnayaka, Dushani Jayawardhana, Manjula Danansuriya, Nalika Gunawardena

**Affiliations:** 1 National Program for Tuberculosis Control and Chest Diseases, Ministry of Health, Colombo, Sri Lanka; 2 Diabetes and Endocrinology Unit, National Hospital of Sri Lanka, Colombo, Sri Lanka; 3 Central Chest Clinic, Colombo, Sri Lanka; 4 Health Informatics Unit, Ministry of Health, Colombo, Sri Lanka; 5 Microbiology Department, National Hospital of Sri Lanka, Colombo, Sri Lanka; 6 World Health Organization Country Office for Sri Lanka, Colombo, Sri Lanka; University of the Witwatersrand, SOUTH AFRICA

## Abstract

End TB strategy by the WHO suggest active screening of high-risk populations for tuberculosis (TB) to improve case detection. Present study generates evidence for the effectiveness of screening patients with diabetes mellitus (DM) for Pulmonary TB (PTB). A study was conducted among 4548 systematically recruited patients over 45 years attending DM clinic at the National Hospital of Sri Lanka. The study units followed an algorithm specifying TB symptom and risk factor screening for all, followed by investigations and clinical assessments for those indicated. Bacteriologically confirmed or clinically diagnosed PTB were presented as proportions with 95% CI. Mean (SD) age was 62·5 (29·1) years. Among patients who completed all indicated steps of algorithm, 3500 (76·9%) were investigated and 127 (2·8%) underwent clinical assessment. Proportion of bacteriologically confirmed PTB patients was 0·1% (n = 6,95%CI = 0·0–0·3%). None were detected clinically. Analysis revealed PTB detection rates among males aged ≥60 years with HbA1c ≥ 8 to be 0·4% (n = 2, 95%CI = 0·0–1·4%). The study concludes that active screening for PTB among all DM patients at clinic settings in Sri Lanka, to be non-effective measure to enhance TB case finding. However, the sub-category of diabetic males with uncontrolled diabetics who are over 60 years of age is recommended as an option to consider for active screening for PTB.

## Introduction

Tuberculosis (TB) remains the world’s deadliest infectious disease. In 2018, TB killed 1.5 million people worldwide [1]. The World Health Organization’s (WHO) End TB strategy aims to reach End TB targets by 2035 while sustainable development goal’s target 3·3 aims at ending the TB epidemic by 2030. Achieving these targets require a global annual decline in the incidence of TB to be 4–5%, although the current decline is 2% [2]. Implementing systematic screening among selected high-risk groups is one recommended intervention to accelerate the decline [3].

Sri Lanka has committed to WHO’s End TB Strategy in 2014 [4]. Estimated burden of TB in Sri Lanka was 64 per 100,000 population in 2018, but the case notification rate for all forms of TB was 40·9 per 100,000 population [5]. This gap between the estimated and the reported TB incidence stands over 4000 [6]. Active screening of high-risk populations, namely prisoners and people living with Human Immuno-deficiency Virus (HIV)/ Acquired Immune Deficiency Syndrome (AIDS) has been made a national policy based on local studies which revealed a TB incidence of 1·7% among prisoners [7] and 9·7% among people living with HIV/AIDS [8].

Research evidence indicate that a patient with diabetes mellitus (DM) is at a three-fold risk of developing active TB compared to a non-diabetic person [9, 10]. This increased prevalence of TB in diabetes may be explained by multiple pathophysiological mechanisms. Phagocytes and lymphocytes are the most important effector cells for containment of TB. Diabetes is known to affect chemotaxis, phagocytosis activation and antigen presentation by phagocytes in response to *mycobacterium tuberculosis* [11]. Impaired chemotaxis of monocytes is evident in patients with diabetes which is not reversed with insulin treatment [11]. There is less activation of alveolar macrophages and decreased production of hydrogen peroxide in tuberculosis patients with diabetes. Furthermore, T cell growth function, proliferation, interferon gamma production is adversely affected in diabetes. Interferon gamma potentiate the nitric oxide dependent intracellular killing activity of macrophages which is important in reducing the bacterial burden of tuberculosis [12]. Higher risk for DM patients to develop TB is discussed in studies conducted among hospital populations [13, 14], as well as among general population cohort studies [15, 16]. Diabetes mellitus is also found to be associated with an increased risk of development, mortality, relapse, recurrence and reactivation of TB [17, 18]. Further, it is associated with a 9-fold risk of treatment failure [19]. Recent evidence indicates that DM is a risk factor for multidrug resistant TB and for delayed sputum smear and culture conversion time as well [20, 21].

Based on these evidence, international health agencies suggested active screening of DM patients for TB as an active case finding strategy [22]. At present, countries like China are actively screening DM patients for TB [23], while countries such as India [24] and Nigeria [25] are planning to introduce active screening based on the evidence of the pilot projects.

At present, Sri Lanka is recognized as a low-burden country for TB [26]. Nevertheless, the gap between the estimated and reported caseloads of TB has been stagnant around 4000 for the past decade [27]. Active screening of patients with DM for Pulmonary TB (PTB) is one among various interventions suggested to improve local case detection to close the gap [6]. The rising burden of DM in Sri Lanka, as evident by the national prevalence of 7.4% in 2015 [28] and of 14.7% in a survey conducted in a suburban district in 2018 [29] is a supporting factor to consider this at-risk population in the country for active screening for TB.

However, the effectiveness of this strategy seems to depend on many variables, including the prevalence of TB in the community. The authors of a systematic review which analyzed bi-directional screening for TB and DM for TB concluded that the yield of active screening of DM patients for TB vary. Hence researchers and experts highlight the need for context specific evidence [30]. Therefore, the objective of the present study therefore, was to generate local programmatic evidence on the proportion of TB among diabetics attending a public DM clinic in an urban setting, to guide the national policy decision on adopting active screening of DM patients for PTB.

## Methods

### Study design, setting, participants and the study size

The study was a hospital based cross-sectional study at the diabetes clinic at the National Hospital of Sri Lanka (NHSL), the largest state hospital in the country. The NHSL diabetic clinic cater for approximately 3600 patients per month from the most urbanized as well as most densely populated district of Colombo.

A registered patient at the DM who was above 45 years was considered as a study unit. Selection of age 45 was based on the age patterns of PTB patients, as PTB is not common among less than 45 years in Sri Lanka. The age pattern of DM clinic attendees also indicated 45 years to be the lower margin of the ages pattern of clinic attendees. Hence, 45 years was taken as the lower age limit to increase the efficiency of the design to detect the PTB patients among DM clinic attendees. There was no upper age limit. Pregnant women, patients who were having difficulties in mobility (as they needed to be transported to a nearby hospital for further investigations) and comprehension were excluded from the study. Size of the sample of study units to be included was calculated based on the number required to estimate the proportion of diabetic patients who are expected to have PTB, using the Lwanga and Lemeshow formula [31] for cross-sectional studies. The estimated proportion of diabetic patients who are expected to have PTB among diabetic patients was considered as 642 case rate per 100,000 patients (0.64%) as reported in an Indian study [23]. The precision of the estimate was taken as 0.015%. The final sample size calculated was 4400. Eligible patients attending the clinic from August 15 to December 14 in 2019 were consecutively recruited to the study after obtaining informed written consent, while data collection continued until March 2020.

### Study variables and data source

**Fig 1:**
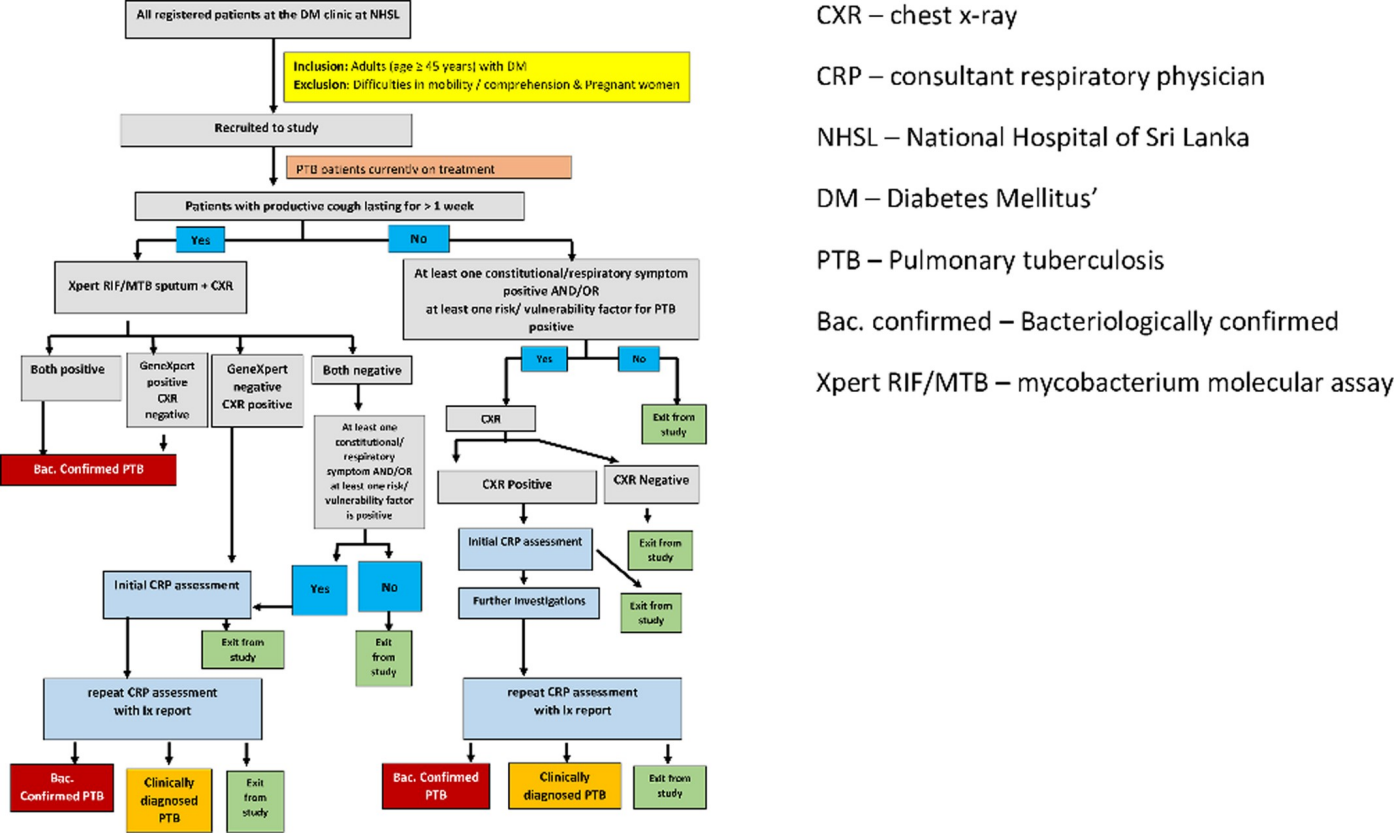
Algorithm used to detect cases of pulmonary TB among attendees of DM clinic.

The algorithm (Fig 1) to be used to detect PTB among the attendees of DM clinic was a stepwise process to direct study units into different care pathways according to pathophysiology-based risk factors for TB among the DM patients. Considering the pathophysiology and complex interactions of TB and DM, we designed the algorithm that any study units with a specified combination of minimum set of risk factors would be directed to investigations to exclude PTB. In addition, the study units were inquired for past history of TB, TB symptoms and risk factors and self-reported information were considered as opposed to requiring documentary evidence. In the absence of robust medial record system with data linkages across health institute to retrieve past medical history of patients, this it was purposely designed so to include all who are likely to have the risk factors being directed to the next step in the algorithm of undergoing investigations to exclude PTB.

This initial screening selected a group of patients who complained of cough more than one week or at least one of the checked risk factors or a symptom to undergo chest x-ray (CXRay), Xpert MTB/RIF testing (a type of molecular testing for TB), culture and clinical assessments based on objective criteria. The algorithms led the study units into three pathways.

#### Pathway A

Those who had been diagnosed with PTB prior to the study and already on treatment for PTB at the time of recruitment. They were not subjected to further investigations but were included as a study unit.

#### Pathway B

Those who had productive cough for more than one week on symptom screening were directed to digital CXRay and to provide an on-the-spot sputum sample for the Xpert MTB/RIF cartridge molecular assay to detect PTB. Xpert MTB/RIF positives were taken as bacteriologically confirmed PTB and were referred for routine care for PTB. Among others, those with CXRay with defined features and those with at least one other self-reported symptom of TB and/or one risk factor were subjected to clinical evaluation and further investigations by a Respiratory Physicians (RP) to rule out clinical PTB.

#### Pathway C

Among patients who had no productive cough at the initial screening but were having at least one self-reported symptom of TB (except productive cough for more than one week, where the patients were directed to pathway B) and/or one risk factor were subjected to CXRay, which is considered as a good screening tool for TB [32]. Those who had a positive CXRay were subjected to clinical assessment and further investigations by a RP to rule out clinical PTB.

The TB symptom and risk factor screening were designed as an electronic interviewer-based questionnaire and was administered by the treating Medical officers in the DM clinic. Participants of Pathway A were inquired into the continuation of treatment and were not subjected to further investigations. Participants of pathways B and C were offered a transport service and were accompanied by a RA to a nearby private hospital for investigations namely digital CXRay and on-the-spot sputum sample for the Xpert MTB/RIF assay. Though the investigation facilities were available in the study setting (NHSL), a private hospital was chosen considering the delays that may occur in getting the investigations and the additional cost to the state institution. Study investigators did random visits to the private hospital to supervise the quality of process and procedures.

Xpert MTB/RIF assay was performed in the Central Chest Clinic—Colombo and in the NHSL microbiology laboratory by the Microbiologists, while cultures were performed at the National Tuberculosis Reference Laboratory at Welisara by consultant microbiologists. Digital CXRay were evaluated by a team of two radiologists independently and blindly. The reporting format and a scoring system was developed by a group of radiologists and respiratory physicians to classify a CXRay as showing evidence of a presence of defined features that may reflect past or active PTB. In the study design we planned to resolve any discrepancies in the reporting between the two radiologists, through discussions to generate a consensus between the two consultant radiologists but there were no discrepancies. DM patients with positive CXRay (with pre-defined features), and those with at least one other self-reported symptom of TB and/or one risk factor of the patients directed to Pathway B and C, were subjected to clinical assessment and further investigations by a RP to rule out clinical PTB. Other relevant investigations and sputum culture of required patients were performed at the National Tuberculosis Reference Laboratory.

Those who reported a positive Xpert MTB/RIF test were considered as bacteriologically confirmed PTB and were referred for routine care and treatment of PTB.

### Outcomes and statistical analysis

Those who were already on treatment for PTB, those who were detected by the present study by a positive Xpert MTB/RIF test or a positive culture were classified as “bacteriologically confirmed PTB” while those with no bacteriological evidence but suggestive clinical features were classified as “clinically diagnosed PTB”. Detection rates of PTB among the total sample as well as among sub-categories were calculated and presented as percentages and respective 95% Confidence Intervals.

### Ethical approval

Ethical approval was obtained from the Ethics Committee, Post Graduate Institute of Medicine, University of Colombo (Registration number ERC/PGIM/2019/134). Informed written consent was obtained from all individual participants included in the study.

## Results

The study recruited 5159 of which, 4548 (88·1%) completed all the relevant steps in the algorithm. The participants were lost along the algorithm at the points of presenting to the interview by Medical Officers (n = 170), not attending for CXRay and/or sputum collection (n = 416), and not presenting for RP assessment (n = 12).

### Basic characteristics of the study units

Mean (SD) age of the sample was 62·5 (29·1) years. Majority (n = 3415; 75·1%) of study participants were between 50–69 years of age, while two thirds (n = 3102; 68·2%) were females. More than half of the study sample (n = 2478; 54·5%) had their last recorded fasting blood sugar levels under controlled levels of less than 130mg/dl (Table 1). At the NHSL DM clinic, HbA1c is done as a routine clinic investigation. HbA1c reports older than 2 months were not considered and recorded as too old data. Reports were available for 4114 (90.4%). However, the last recorded HbA1c levels indicated good glycaemic control (< 8) only among 31·9% (n = 1451). Comparison of the proportions of males and females with good glycemic control (HbA1c < 8) (males 41·2%; females 69·6%) and the very poor glycaemic control (HbA1c≥11) (males 10·0%; females 20·9%) indicated that the females were having worse DM control compared to males.

**Table 1:** Distribution of basic socio-demographic and illness-related characteristics of the study sample.

Characteristic	Number	%
**Age**		
45–49 years	311	6·8
50–59 years	1603	35·2
60–69 years	1819	39·9
≥70 years	815	17·9
**Sex**		
Male	1446	31·8
Female	3102	68·2
**Ethnicity**		
Sinahalese	2991	65·8
Sri Lankan Tamil	793	17·4
Indian Tamil	42	0·9
Muslim	676	14·9
Burgher	15	0·3
Maley	31	0·7
**Highest educational qualification**		
Never been to school	234	5·1
Up to grade 5	655	14·4
Grade 5–11	1530	33·6
O/L completed	1299	28·6
Grade 12–13	52	1·1
A/L completed	673	14·8
Undergraduate	54	1·2
Post graduate	51	1·1
**Status of occupation**		
Never employed	1387	30·5
House maker/ house wife	1130	24·8
Currently occupied	1242	27·3
Retired	789	17·3
**Average monthly family income** (LKR)		
< 20,000	2373	52·2
20,000–49,999	1613	35·5
50,000–99,999	169	3·7
≥100,000	30	0·7
Refused/ unknown	363	8·0
**Duration of DM**		
<1 year	2525	55.5
1–5 years	1955	43.0
6–10 years	17	0.4
>10 years	51	1.1
**Glycaemic control**		
HbA1C ≤ 8	1451	31.9
HbA1c > 8	2663	58.6
HbA1C reports older than 2 months/ reports not available	434	9.5
**Drugs for DM**		
Oral hypoglycaemics only	2564	56.4
Oral hypoglycemics + Insulin	1733	38.1
Insulin only	240	5.3
Data not available	11	0.2

### Results of screening for symptoms of TB and risk factors

The symptom screening tool inquired study units on the presence of productive cough of at least one-week duration. Cough for any duration was present among 431 (9·5%) of the 4548 study participants, with 315 (6·9%) complaining of cough more than one-week duration.

Of all 4548 study units, approximately one fourth (n = 1123, 24·7%) complained of at least one symptom which could be related to PTB. The common symptoms were on and off difficulty in breathing (n = 514), loss of appetite (n = 456), and night sweats (n = 393).

Information through risk factor screening revealed 4048 (89·0%) with at least one vulnerability factor related to PTB. Most common was uncontrolled DM of HbA1C >8 (n = 2898, %), followed by elderly age (n = 2380, %) and immunocompromised status, including chronic diseases such as chronic kidney disease and cancer (n = 435, %). Least common was use of narcotic drugs (n = 8, %), imprisoned or having worked in a prison (n = 16, %) and malnutrition with BMI <18.5 (n = 53, %).

Most females (42·8%) were in the overweight category with a BMI 25·0–29·0, while most males (46·9%) had recommended BMI of 18·5–24·9. Among the other risk factors inquired, past history of TB was reported by 141 (3.1%), while history of close contact with a TB patient within past two years was reported by 106 (2.3%).

### Results by pathways of the algorithm

Of the 4548 study participants, 13 (0·3%) had been diagnosed prior to the study and were already on treatment for PTB at the time of recruitment.

As shown in Table 2, Pathway B was for those who had productive cough for more than one week (n = 315). Of the 315 eligible for MTB/RIF testing and digital CXRay, 292 (92·7%) underwent MTB/RIF testing and 289 (91·7%) underwent digital CXRay.

**Table 2:** Distribution of the study units who completed the study by investigations and assessments conducted (n = 4548).

Investigations/ assessments	Number (%)	Positive results
**Pathway B**	315 (100.0)	
• CXR	289	9
• Xpert MTB/RIF	292	3
• Consent withdrawn	23	--
**Pathway C**	4661 (100.0)	
• CXR	3023	65
• Consent withdrawn	393	---
**Further investigations by CRP from pathways B and C**	128 (100.0)	
• Xpert MTB/RIF	25 (0·5)	1
• Culture	30 (0·6)	3
• Could not be contacted	12	---
**Indication for the CRP referral (n = 128)**		
• Cough + positive CXRay, negative Xpert MTB/RIF	7 (5·4)	1
• Cough + negative CXRay & Xpert MTB/RIF + positive risk/ vulnerability factor	56 (43·7)	0
• No cough BUT positive CXRay and/or at least one positive constitutional symptom and/or at least one positive risk/ vulnerability factor	65 (50·8)	2

CXray–chest x-ray.

Xpert MTB/RIF–GeneXpert testing.

PTB–Pulmonary tuberculosis.

Pathway C was for those who had no productive cough but had either at least one other symptom and/or one risk factor (n = 3124). Of the 3124, 3023 (96·8%) were subjected to digital CXRay.

All in all, of the 4548 study units, 3500 (76·9%) were subjected to further investigations. Among the study units, the number underwent Xpert MTB/RIF at any point of either pathway was 317 (6·9%) and the corresponding number who underwent CXRay was 3312 (72·8%). Out of the 3312 CXRay, only 74 (2·2%) were reported as having defined features by the consultant radiologist according to the laid down criteria.

The number of study units eligible for RP assessment from both pathways B and C was 128, of which 12 defaulted. The indication for the CRP referral is shown in Table 2. As part of the RP clinical evaluation, Xpert MTB/RIF was performed on 25 study units while culture was performed on 30 study units (Table 2).

**Detection of PTB cases among the study population.** Of all 4548 study participants, six (06) patients were detected to have PTB as a result of active screening by the present study giving a proportion of PTB among diabetes clinic attendees as 0·001 (6/4548). As indicated above the proportion of study units who had been diagnosed prior to the study and was on treatment for PTB at the time of recruitment was 0·003 (13/4548). All of them were included to the category of bacteriologically confirmed PTB. The proportions of PTB patients among selected sub-categories of the study sample were also analyzed (Table 3).

**Table 3:** Proportions of pulmonary tuberculosis patients detected by the study.

Sub-category	Number of PTB patients	Proportion	%	95% CI
• Bacteriologically confirmed as PTB detected by the present study	6	0·001	0·1	0·0%–0·3%
• Bacteriologically confirmed as PTB and had been prescribed on treatment f or PTB at the time of recruitment	13	0·003	0·3	0·2%–0·5%
• Clinically diagnosed PTB	0	0·000	0·0	---
• Confirmed as no PTB	4529	0·996	99·6	---

The male: female ratio was 2:1 among the PTB patients detected by the present study. Four (66.7%) of the patients were in their fifties. All six patients (100.0%) had poor glycaemic control indicated by HbA1c ≥ 8. Five of them (83.3%) had body mass index within the normal rage while the other patient (16.7%) was overweight (Table 4). Further analysis of data revealed a percentage of PTB patients (0.3%, 95% CI = 0·10%– 0·70%) among the males. Among females it was 0·06% (95% CI = 0·00% - 0·20%). The same percentage among sub-categories of ≥ 60 years of age and HbA1c≥ 8 were 0.002 each. Males ≥ 60 years of age with HbA1C ≥ 8 reported the highest percentage of 0.4% (95% CI = 0·05%-1·40%).

**Table 4:** Distribution of socio-demographic and illness-related characteristics of patients with pulmonary tuberculosis detected.

Characteristic	Patient #1	Patient #2	Patient #3	Patient #4	Patient #5	Patient #6
Age	51	59	56	68	84	57
Sex	Male	Female	Female	Male	Male	Male
Monthly family income (LKR)	20,000–50,000	< 20,000	20,000–50,000	20,000–50,000	20,000–50,000	20,000–50,000
Body Mass Index	23	24	22	22	20	28
Duration of DM	1year 6 months	8 months	10 months	2years 6 months	2years 3 months	5 months
Medication for DM	OHA only	OHA+ Insulin	OHA+ Insulin	Insulin only	OHA only	OHA+ Insulin
Previous month Fasting blood sugar mg/dl	95	100	136	173	111	126
Last recorded HbA1c	11	10	15	13	9	11
Productive cough	No	Yes	Yes	Yes	No	Yes
Duration of cough	Not applicable	> 2 weeks	> 2 weeks	> 2 weeks	Not applicable	More than a month
Care pathway	C	B	B	B	C	B
Results of investigations						
• Xpert MTB/RIF	Positive	Positive	Positive	Positive	Negative	Negative
• CXRay	Positive	Positive	Negative	Positive	Positive	Negative
• Culture	Positive	Not done	Not done	Not done	Positive	Positive

CXray–chest x-ray.

DM–diabetes mellitus.

IHD–ischaemic heart disease.

OHA–oral hypoglycaemic agents.

Xpert MTB/RIF–GeneXpert testing.

Table 4 illustrates the socio-demographic and illness-related characteristics of patients detected through active screening. Four out of six patients were males, and all of them were from economically disadvantaged families. The glycaemic control, as depicted by the HbA1c values, were poor among of all them.

## Discussion

### Key findings

The proportion of PTB detected by active screening among all diabetes clinic attendees was 0·001 (6/4548). All of them were included in the category of bacteriologically confirmed PTB. The proportions of PTB patients detected among sub-categories of the study sample revealed a percentage of PTB patients (0.3%, 95% CI = 0·10%– 0·70%) among the males. Among females it was 0·06% (95% CI = 0·00% - 0·20%). The same percentage among sub-categories of ≥ 60 years of age and HbA1c≥ 8 were 0.002 and 0.002 respectively. Males ≥ 60 years of age with HbA1C ≥ 8 reported the highest percentage of 0.4% (95% CI = 0·05%-1·40%).

### Interpretation

Although active screening of DM patients for PTB is proposed as a measure to close the gap between estimated and reported PTB cases [22], the effectiveness of this strategy depends on many factors. The prevalence of PTB among DM is one such important factor. The number of diabetics needed to screen to find one extra case of PTB is directly related to the local TB prevalence. The yield of screening increases with the prevalence of TB in the locality [30].

However, even countries with high burden of TB report different results for the number of TB patients detected actively through screening of DM patients. This can be owed to the programmatic issues related to implementing the programme of screening. China and Marshall Islands are countries where both TB and DM are highly prevalent and have reported high rates of detection of TB patients through active screening among DM. In China, one study reported a TB prevalence of 342.7 per 100,000 persons with DM [33] while another study reported the TB prevalence among DM as 102 per 100,000 [34]. These are higher rates when compared with the TB prevalence among the local general population of 42.8 per 100,000 persons [34]. Similarly, a study from the Republic of the Marshall Islands reported detection of 11 new TB cases after actively screening 353 DM patients, at a rate of 3116 per 100,000 DM patients [35] which is much higher than the prevalence of 483 of TB among general population.

On the other hand, India, also a country with high caseloads of TB and DM reports detecting lower rates of TB through active screening of DM patients. One study revealed only 18 patients with TB through active screening of a group of 11,691 DM patients [24] with an incidence rate of 153.9 per 100,000 DM patients, whereas the incidence rate of TB among the general population in India is 199 per 100,000 [36]. Similarly, another Indian study reported not being able to detect a single case of TB through active screening of 630 DM patients, whose median age was 60 years and the median HbA1c level was 8.7% [37]. It is interesting to note that all these studies have used similar screening methods with initial symptom checklists followed by sputum examination of Xpert MTB/RIF and acid-fast bacilli (AFB) testing and CXRay to diagnose TB patients.

Sri Lanka is a country with low prevalence of PTB. Its prevalence is estimated by WHO as 0·06% (95% CI = 0.05%-0·08%) [5]. Sri Lanka records a prevalence of DM of 7.3 [38] which is comparable to the other Asian countries [39]. The present study showed that the prevalence of screened PTB among the DM population to be 1·7 times higher than the PTB prevalence in the general population. When considering the subgroup of diabetic males >60 years of age with HbA1C >8 the prevalence of screened PTB was seven times higher than the PTB prevalence in the general population. At present, two specific populations are recommended for active screening of PTB in Sri Lanka. They are prison inmates and people living with HIV/AIDS. The proportion of PTB patients detected through active screening of prison inmates is 1·6% (95%CI = 1·4%-2.1) [7] and of people living with HIV/AIDS is 9.7 (95% CI = 6·9%–12·9%) [8]. Accordingly, the screened PTB was 26 and 161 times higher than the PTB prevalence in the general population among prison inmates and among people living with HIV/AIDS, respectively.

Accordingly, the highest prevalence of screened PTB (0.4%) is among the sub-category of diabetic males with poor glycaemic control. Male sex [27, 40] and poor glycemic control [41] are well-evident factors associated with the risk of developing TB among DM patients. Authors of a review article [10] recommended active screening among uncontrolled diabetics and diabetic children with recent exposure to a TB patient, rather than mass screening of all DM patients. Similarly, an Australian nation-wide cohort study [16] concluded that DM alone does not warrant active screening of patients for TB.

### Strengths and limitations of the study

The present study used an algorithm designed to direct study units into different care pathways based on pathophysiologically explainable risk factors for TB among the DM patients. Considering the pathophysiology and complex interactions of TB and DM, we designed the algorithm that any study units with a specified combination of minimum set of risk factors would be directed to investigations to exclude PTB. For instance, after filtering out the patients who are currently on treatment for TB, the study units were inquired about having productive cough for a duration of one week or more, rather than two weeks which is typical for TB. Thereafter, study units with even a single pathophysiology-based risk factor for TB were directed into different care pathways considering the pathophysiology and complex interactions of TB and DM. As a result, even a study unit with a minimum set of risk factors would be directed to investigations to exclude PTB. In addition, DM patients above 45 years were recruited as TB is more prevalent among >45 years in Sri Lanka [27]. The age pattern of DM clinic attendees also indicated 45 years to be the lower margin of the ages pattern of clinic attendees. Hence, 45 years was taken as the lower age limit to increase the efficiency of the design to detect the PTB patients among DM clinic attendees.

The main limitation of the study is that it was conducted only in one DM clinic which caters for a group of patients belong to middle and lower socio-economic groups in the country. However, considering the fact that PTB is known to be common among such socio- economic groups in Sri Lanka [42], the estimate of the proportion of PTB among DM is not likely to be an underestimate. In addition, majority of the study sample was females, whereas PTB is commoner among males in Sri Lanka [27]. Anyway, the majority of attendees of any public DM clinic in the country would comprise females [43, 44]. Hence it could be safely assumed the estimations of this pragmatic study to be realistic, if active screening was to be conducted in real settings. DM patients also having chronic NCDs such as hypertension and ischemic heart disease may attend general medical clinics at the NHSL rather than the DM clinic. Unfortunately, the proportion of such patients cannot be calculated, as government hospital clinics in Sri Lanka do not possess a information system that allows estimation of this parameter. This can be considered as a limitation of this study.

## Conclusions

Active screening for PTB among all DM patients at clinic settings in Sri Lanka, a country with low burden for TB, is found to be non-effective measure to enhance TB case finding, given the very low prevalence rates of PTB among DM clinic attendees. However, the sub-category of diabetic males with uncontrolled diabetics who are over 60 years of age is an option to consider for active screening for TB. This requires further studies capturing different local settings such as DM clinics at peripheral hospitals, to arrive at a conclusion on the effectiveness of actively screening DM patients to close the gap in TB estimated and reported numbers.

## Supporting information

S1 Dataset (XLS)

S1 Annex Questionnaire to assess the proportion of patients with pulmonary tuberculosis among patients attending the diabetes clinic at National Hospital of Sri Lanka. (DOCX)
