# Supplementary material for: Active screening of patients with diabetes mellitus for pulmonary tuberculosis in a tertiary care hospital in Sri Lanka
Source: PLoS One. 2021 Apr 8;16(4):e0249787. doi: 10.1371/journal.pone.0249787 (PMC8031956; doi:10.1371/journal.pone.0249787)
Supplement: S1 Annex — (DOCX) [file pone.0249787.s002.docx]

**S1 Annex**

**Questionnaire to assess the proportion of patients with pulmonary tuberculosis among patients attending the diabetes clinic at National Hospital of Sri Lanka**

| Date |  |
| --- | --- |
| Diabetes Clinic No. |  |
| Index Number |  |

***Instructions to the interviewer: Read out the following paragraph to the patient.***

Thank you very much for taking part in this research.

As described to your earlier also, this study is done by the Health Ministry and the World Health Organization. It is well known that those with diabetes have a higher chance of suffering also from tuberculosis which often is symptomless and hidden. World Health Organization advocates that those who are suffering from diabetes with specific other features are routinely subjected to screening of tuberculosis by performing investigations to check for TB. If found to have TB, there are very effective treatment that can cure the diseases.

This survey is to check whether those attending this clinic have pulmonary tuberculosis. As a result of participation, you will get to know about your TB status, and if you are found to have TB, you will be treated for free of charge until you are cured.

You will be asked few questions now, and if I feel like you might be having TB depending on your answers, you will be sent for a chest X-ray and sputum examination at a private hospital, at the end of this interview. You will be transported to this private hospital by us, and we will be paying for your chest X-ray and sputum examination. After finishing the tests there, you will be transported back to here, and by the time you come back, your diabetic drugs will be ready. So, you can collect your medicine without waiting and go home.

The results of the tests will be informed to you at the next diabetes clinic visit. If you are found to be having tuberculosis or needing further evaluations you will be directed to them.

| **Section I**  **Patient’s personal details** |
| --- |

- 1. National ID card number:
  2. Address:
  3. Your telephone numbers:

| Land number |  | Mobile number |  |
| --- | --- | --- | --- |

- 1. Another contact number of a family member:

| Land number |  | Mobile number |  |
| --- | --- | --- | --- |

- 1. Date of birth: …………………………………………..
  2. Age in complete years: ………………………………
  3. Sex: ………………………..
  4. What is your ethnicity?

| Sinhalese |  | Sri Lankan Tamil |  | Indian Tamil |  | Muslim |  | Burgher |  |
| --- | --- | --- | --- | --- | --- | --- | --- | --- | --- |
| Maley |  | Other |  |  |  |  |  |  |  |

- 1. What is your marital status?

| Never married |  | Married |  | Living together |  |
| --- | --- | --- | --- | --- | --- |
| Separated |  | Divorced |  | Widowed |  |

- 1. What is your highest educational qualification?

| Never been to school |  | Up to grade 5 |  | Grade 5 - 11 |  |
| --- | --- | --- | --- | --- | --- |
| O/L completed |  | Grade 12-13 |  | A/L completed |  |
| Undergraduate |  | Postgraduate |  |  |  |

- 1. What is your status of occupation?

| Unemployed |  | House maker/ house wife |  | Currently occupied |  |
| --- | --- | --- | --- | --- | --- |
| Retired |  |  |  |  |  |

- 1. What is your average family income per month? ……………….. LKR

| **Section II - Illness related information** |
| --- |

***Instructions to the interviewer:***

**Ask the questions and wherever available also use the diabetic clinic book and any other medical records available with the patient to verify the reported information.**

2.1: When were you first diagnosed to have diabetes mellitus? …………….. months ago

2.2: what are the medications you are currently on? (Ensure the accuracy of the information by cross checking with the clinic book).

| Oral hypoglycaemics |  | Insulin |  | Both |  | On diet control only |  |
| --- | --- | --- | --- | --- | --- | --- | --- |

|  | mg/dl | Date |  |
| --- | --- | --- | --- |

2.3: What was your last Fasting Blood Sugar?

(cross check with medical records)

| Value |  | Date |  |
| --- | --- | --- | --- |

2.4: What was your last HbA_1_C?

(cross check with medical records)

| Value |  | Date |  |
| --- | --- | --- | --- |

2.5: What is your last recorded Body Mass Index? (cross check with medical records)

2.6: Have you **ever** undergone screening for tuberculosis by providing a sputum sample or by doing a Chest x -ray? Yes / No

2.7: If yes, when was it last done? …….. months / …. Years ago

| TB detected |  |
| --- | --- |
| TB not detected |  |

2.8: What was the result?

| Yes |  | Go to Section III |
| --- | --- | --- |
| No |  | Go to Section IV |

2.9 Are you currently on treatment for tuberculosis?

| **Section III - Information on those under treatment for TB** |
| --- |

***Instructions to the interviewer:***

If the patient is currently on treatment for tuberculosis, ask for any medical records on tuberculosis management like the DOT record card or clinic book or any other medical records available with the patient to retrieve necessary information in question 3.1 and 3.2.

If the patient does not have any medical records related to tuberculosis, inform him/ her that he/she will be contacted by the research team through phone to collect information. Also ensure the patient’s contact details are accurately documented.

3.1 Which chest clinic is the patient been followed up at for tuberculosis?

(E.g.: Colombo municipal council/ Colombo district chest clinic/ Gampaha district chest clinic etc. If the patient does not know, please mention as ‘don’t know’)

| Name of the Chest clinic |  | Cross checked with medical records |  | Medical records not available |  |
| --- | --- | --- | --- | --- | --- |
| Does not know |  |  |  |  |  |

3.2 Type of tuberculosis (from the clinic book)

| Pulmonary TB |  | Extra pulmonary TB |  | Cross checked with medical records |  | Medical records not available |  |
| --- | --- | --- | --- | --- | --- | --- | --- |

| **Section IV**  **Screening for TB** |
| --- |

| Yes |  | Go to question no. 4.2 |
| --- | --- | --- |
| No |  | Go to question no. 4.4 |

4.1 Do you have cough at present?

| Yes |  | Go to question no. 4.3 |
| --- | --- | --- |
| No |  | Go to question no. 4.4 |

4.2 Do you bring out phlegm when you are coughing at present?

| Less than a week |  |
| --- | --- |
| 1-2 weeks |  |
| More than 2 weeks |  |
| More than a month |  |
| Other (specify) | |

4.3 If yes, duration of the productive cough?

| ***Instructions to the interviewer:***  **Based on the above responses, mark the relevant cage for this respondent.** | | | | |
| --- | --- | --- | --- | --- |
| **Patient is having a productive cough more than one week?** | **Yes** |  | **No** |  |

4.4 Read out the listed symptoms one by one and mark the patient’s response as yes or no. As you do, also allocate the appropriate score for each item. Calculate the total score at the end.

| **Symptom** | **Yes** | **No** | **Score allocation** | **Patient’s score** |
| --- | --- | --- | --- | --- |
| Sweating at nights even on days which are not very warm and when no one else in the house is complaining of seating **on most of the days, within past one month** |  |  | If yes 🡪 2  If no 🡪 0 |  |
| Noticed that you are losing weight or getting thinner in spite of any attempts to purposely lose weight, **within past one month** |  |  | If yes 🡪 2  If no 🡪 0 |  |
| Has been having slight fever/ feverish feeling on **most of the days, within past one month** |  |  | If yes 🡪 2  If no 🡪 0 |  |
| Appetite for food has been low **on most of the days, within past one month** |  |  | If yes 🡪 2  If no 🡪 0 |  |
| Coughed up blood/ blood stained sputum **at least once during the month, within past one month** |  |  | If yes 🡪 2  If no 🡪 0 |  |
| Difficulty of breathing/ shortness of breath **on most of the days, within past one month** |  |  | If yes 🡪 2  If no 🡪 0 |  |
| **Total score** |  |  |  |  |

4.5 Mark the patient’s response as yes or no and allocate the appropriate score for each item. Calculate the total score at the end.

| **Symptom** | **Yes** | **No** | **Score allocation** | **Patient’s score** |
| --- | --- | --- | --- | --- |
| Have you **ever** been diagnosed as having tuberculosis in lungs or any other organ of the body in the past? |  |  | If yes 🡪 4  If no 🡪 0 |  |
| Was any one living in the same house (can be a family member/ relative/ boarder etc.) or work place as you were, diagnosed with TB in the lungs **within last 2 years?**  (a clue to indicate TB in the lung is being diagnosed after a sputum testing) | What is that patient’s relationship to you?  (e.g.: father/ mother/ sister/ brother/ grandparent/ work mate etc) |  | If yes 🡪 4  If no 🡪 0 |  |
| Have you **ever** been or are you a health care worker (doctor, nurse, attendant, health assistant, lab related, pharmacy or any other)? |  |  | If yes 🡪 4  If no 🡪 0 |  |
| Have you smoked tobacco in any forms of cigarrates, beedi, cigars **within the past year?** |  |  | If yes 🡪 4  If no 🡪 0 |  |
| Are you suffering from any of the following illnesses/conditions?   1. HIV/AIDS 2. Chronic kidney disease 3. Undergone any organ transplant 4. Cancer 5. Hematological malignancy 6. Prednisolone 20mg/day or equivalent **continuously for more than 2 months** 7. Use of any other immunosuppressants **continuously** **for more than 2 months** |  |  | If yes to any 🡪 4  If no 🡪 0 |  |
| **Within the last 2 years**, have you been imprisoned or have you worked in prisons? |  |  | If yes 🡪 4  If no 🡪 0 |  |
| Have you lived in the same place OR worked in the same place with foreigners **within the past 2 years?**  If yes ask for the country and consider as yes if the countries are India/ Bangladesh/ China/ Pakistan (please refer to the last page) |  |  | If from any country mentioned 🡪 4  If not 🡪 0 |  |
| Have you worked in mines, construction work, stone crushing, silica /porcelain /pottery /quartz / slate pencil corporation **within last 2 years?** |  |  | If yes 🡪 4  If no 🡪 0 |  |
| Have you **ever used** oral or injectable narcotic drugs? Or are you **currently injecting** narcotic drugs? |  |  | If yes 🡪 4  If no 🡪 0 |  |
| Do you consume any variety of alcohol on **most of the days in the past one month within at least** following amounts per day of drinking?  **Males**   - Wine 150 ml - Arrack/ Whisky/ other hard liquor – 50 ml - standard beer 1 can   **Females**   - Wine 75 ml - Arrack/ Whisky/ other hard liquor – 25 ml - standard beer 1/2 can |  |  | If yes 🡪 4  If no 🡪 0 |  |
| ***Instructions to the interviewer:***  **Please add scores for the followings using the answers given by the patient before*.*** | | | | |
| Look at patient’s **last available** FBS or HBA_1_C |  |  | FBS >160mg/dl or  HBA_1_C >8 🡪4  If not 🡪 0 |  |
| Look at patient’s last recorded BMI. If BMI is not available, calculate BMI using the formula = Weight (kg)  Height (m)^2^ |  |  | If < 18.5 🡪4  If not 🡪0 |  |
| How old is the patient? |  |  | >60 yrs 🡪 4  <60 yrs 🡪 0 |  |
| **Total** |  |  |  |  |

| **Instruction to the interviewer:**  Please refer to the algorithm to decide on the next course of actions for the patients with following information.   - Does the patient has productive cough for more than one week (question no: 4.3) - Total score for the question no. 4.4 - Total score for the question no. 4.5 |
| --- |

**Criteria to decide on the care pathway**

| Answer to question 2.9 | 🡪 | Yes  (currently on treatment for TB) | 🡪 | Pathway A  No further investigations |
| --- | --- | --- | --- | --- |
| Answer to question 4.3 | 🡪 | more than a week cough | 🡪 | Pathway B  Refer for Chest Xray and sputum Xpert tests |
| Total score for table 4.4 | 🡪 | 2 or more | 🡪 | Pathway C  Refer for Chest Xray |
| Total score for table 4.5 | 🡪 | 4 or more | 🡪 | Pathway C  Refer for chest Xray |
